# A rare case of sarcoidosis involving the middle turbinates: an incidental diagnosis

**DOI:** 10.1186/1746-1596-1-44

**Published:** 2006-11-21

**Authors:** Seyra Erbek, Selim S Erbek, Emine Tosun, Ozcan Cakmak

**Affiliations:** 1 Department of Otorhinolaryngology, Baskent University Faculty of Medicine, Ankara, Turkey; 2 Department of Pathology, Baskent University Faculty of Medicine, Ankara, Turkey

## Abstract

**Background:**

Sarcoidosis is a chronic, systemic granulomatous disease of unknown etiology that features noncaseating granulomas in many body regions. Sinonasal involvement is rare but is also suspected to be underreported.

**Case presentation:**

We present the case of a 39-year-old woman who was incidentally diagnosed with isolated sarcoidosis involving the middle turbinates. Histopathologic examination of resected concha bullosa material and an extensive panel of diagnostic tests revealed a diagnosis of isolated sarcoidosis. Since no systemic manifestations were detected, topical corticosteroid (nasal spray) was administered in the postoperative period. Throughout the 12 months after surgery, the patient remained free of symptoms and all nasal endoscopy examinations were normal.

**Conclusion:**

Although isolated nasal involvement of sarcoidosis is rare, otorhinolaryngologists should consider this condition in a differential diagnosis for sinonasal complaints.

## Background

Sarcoidosis is a systemic granulomatous disease of unknown cause. The epidemiology hints at both genetic factors and environmental agents [1]. Sarcoidosis most frequently affects young and middle-aged women and may involve a variety of sites, including the lungs (most common), skin, liver, eyes, spleen, peripheral lymph nodes and neural structures. Approximately 10% to 15% of patients with sarcoidosis exhibit otorhinolaryngologic manifestations, but these are rarely the presenting disorders [2]. The otorhinolaryngologic signs and symptoms of sarcoidosis are not specific and can mimic other more common disorders. We report a case of isolated sarcoidosis of the middle turbinates that was diagnosed after histopathological examination of resected concha bullosa material.

## Case presentation

**Figure 1:**
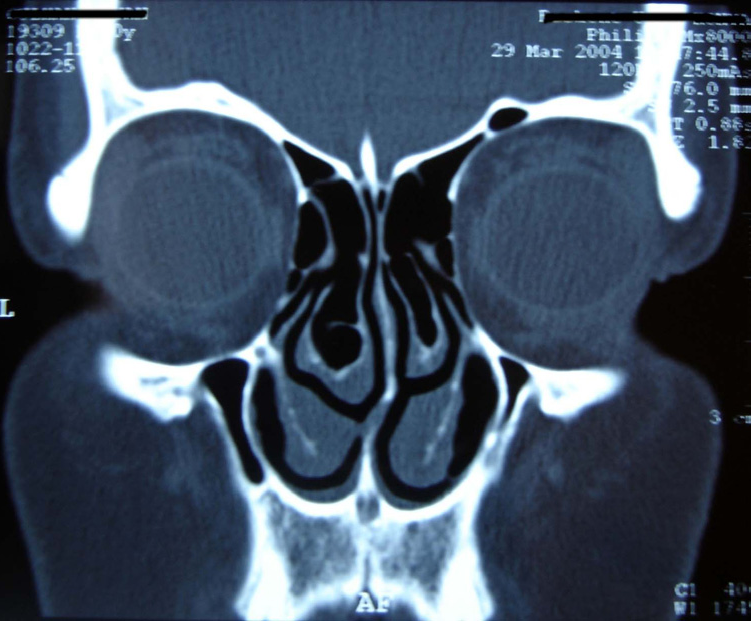
CT scan shows septal deviation and bilateral concha bullosa.

A 39-year-old woman was referred to our clinic with symptoms of headache, nasal obstruction and recurrent epistaxis. These problems had existed for 8 months. Otorhinolaryngologic examination revealed septal deviation and a hypertrophic left middle turbinate. Computed tomography (CT) of the paranasal sinuses revealed septal deviation and bilateral concha bullosa (Figure 1).

**Figure 2:**
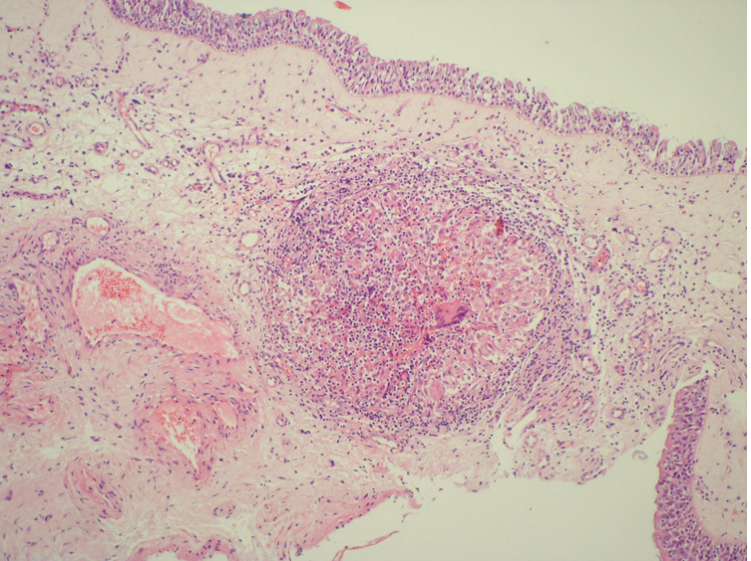
The view of granuloma under the respiratory epithelium of middle turbinate (HE-staining, original magnification ×100).

The patient was placed under general anesthesia and underwent septoplasty and bilateral concha bullosa resection via lateral laminectomies. Histopathologic evaluation of the concha bullosa material from both the left and right sides revealed chronic inflammation and noncaseating granulomas. The granulomas were composed of epithelioid cells and a few Langerhans giant cells, and thus indicated various possible granulomatous diseases, including sarcoidosis (Figure 2).

Consults were done in the departments of internal medicine, pulmonology and ophtalmology. Physical examinations, CT of the abdomen and chest, and pulmonary function tests were normal. Bronchoalveolar lavage was performed; cytology of fluid was normal, and stain as well as culture for *Mycobacterium tuberculosis *was negative. Complete blood cell count, erythrocyte sedimentation rate, electrolytes, liver and kidney function tests, serology for syphilis (VDRL), antineutrophil cytoplasmic antibody were obtained in the patient, and all results were normal. Tuberculosis testing with purified protein derivative (PPD) revealed an induration of 13 mm, and the serum level of angiotensin-converting enzyme (ACE) was 81.9 U/L. Based on all findings, the diagnosis was isolated sarcoidosis of the middle turbinates.

Topical corticosteroid nasal spray was administered in the postoperative period. Throughout the 12 months after surgery, the patient remained free of symptoms and all nasal endoscopy examinations were normal.

## Discussion

Sarcoidosis rarely involves the head and neck region and can be a diagnostic challenge for the otorhinolaryngologist. Involvement of nasal mucosa was first reported by Boeck in 1905 [3]. In patients with sarcoidosis, sinonasal involvement may develop, but the disease is rarely isolated to this area [4]. McCaffrey and McDonald [5] reviewed the records of 2319 patients diagnosed with sarcoidosis and found nasal mucosa involvement in 17 (less than 1%) of these cases. Wilson et al. [4] noted nasal involvement confirmed by biopsy in 21 (2.8%) of 750 patients with sarcoidosis. A recent report by Zeitlin et al. [6] stated a 4% incidence of nasal involvement in 159 patients with sarcoidosis. Those authors also noted that the actual incidence might be much higher. The most frequent sites of nasal involvement are the nasal septum and inferior turbinate, followed by the paranasal sinuses, nasal bone and cartilage, and subcutaneous tissues is the region [7]. To the best of our knowledge, no cases of isolated sarcoidosis of the middle turbinates have been reported to date [2-7]. Although there was no evidence of systemic involvement, this case might be an early manifestation of sarcoidosis, and should be followed-up for a long period.

The symptoms of nasal sarcoidosis are nonspecific. Nasal obstruction is the most frequent symptom, and others include postnasal drip, epistaxis, headache and recurrent sinus infections. Physical examination may reveal dry, friable mucosal lesions involving the septum and inferior turbinates, thick discharge and crusted polypoid tissues. Our patient's left middle turbinate was hypertrophic but no mucosal changes or crusting was observed during nasal endoscopy.

Histopathologic examination of a nasal biopsy is required for definitive diagnosis of nasal involvement of sarcoidosis. In some cases, this is the first evidence of systemic disease. Histologically, affected tissues exhibit multiple epithelioid cell granulomas that are organized collections of mature mononuclear cells. Hyaline fibrosis, leukocyte infiltration, necrosis, and refractile structures within epithelioid cells may also be present [8]. The epithelioid cells secrete a lot of cytokines and mediators, including ACE [8]. Thus, serum ACE level is elevated in 80% to 90% of patients with sarcoidosis [2]. Our patient also exhibited elevated serum ACE. This sign is suggestive of sarcoidosis but is not a diagnostic biochemical marker. Further diagnostic tests are usually necessary to exclude other granulomatous disorders, such as tuberculosis, aspergillosis, actinomycosis, Wegener's granulomatosis, Churg-Strauss syndrome, lymphoma [3]. In our case, we performed all indicated tests and examinations, and the results ruled out other granulomatous diseases.

In patients with sarcoidosis CT of the paranasal sinuses can show various types of lesions. These include multiple mucosal nodules (typically associated with various sinus opacities and inflammatory rhinitis), septal perforation, and destruction of nasal bone and/or cartilage [9]. However, sites of opacification on CT are nonspecific radiologic findings, and accurate recognition of granulomas or nodules requires considerable expertise [3]. Our patient's paranasal CT scan revealed bilateral concha bullosa but no evidence of sinusitis or any lesions in the nasal mucosa.

The clinical course of sarcoidosis is highly variable. There is a high incidence of recurrence, and a high rate (60%–70%) of spontaneous remission as well [1]. Krepsi et al. [10] proposed a staging system for sinonasal sarcoidosis. Patients in stage 1 have mild reversible disease without paranasal sinus involvement. Those in stage 2 have moderate potentially reversible disease with sinus involvement, and stage 3 is characterized as severe, irreversible disease. According to these descriptions, our patient had stage 1 sinonasal sarcoidosis.

Oral corticosteroids are the main treatment for systemic sarcoidosis [1]. Cytotoxic agents (methotrexate and azathioprine), cyclophosphamide, chlorambucil, and anti-malarial drugs (chloroquine and hydroxychloroquine) are the alternative drug therapies for this patient group. The optimum treatment for nasal sarcoidosis depends greatly on the location and severity of the lesions. In patients with involvement of nasal structures or airways, topical or inhaled steroids can be used to avoid the complications that can occur with systemic corticosteroids [3,6,7]. Use of additional intralesional steroid injections in cases of sinonasal sarcoidosis has also been reported [2,10,11]. Systemic steroids are indicated in cases where symptoms are severe and the clinical course is highly destructive [3,6,7,10].

Marks and Goodman [12] suggested that surgery is indicated when medical treatment fails. They reported excellent short-term results and symptomatic improvement in all patients after surgery, but noted that the long-term results were less encouraging. Endoscopic sinus surgery (ESS) is effective for those few patients who develop nasal obstruction or chronic sinusitis due to anatomic blockage from sinonasal sarcoidosis lesions [13]. Kay and Har-El [13] concluded that although ESS does not eradicate the disease or prevent recurrence, it markedly improves quality of life by relieving symptoms and reducing the need for systemic steroids. Our patient underwent septoplasty and bilateral endoscopic concha bullosa resection via lateral laminectomies. She showed no disease progression and had no recurrence of complaints during 12 months of follow-up.

Nasal involvement of sarcoidosis is rare, but otorhinolaryngologists should consider this condition in differential diagnosis of sinonasal complaints. Nasal topical steroid application can control the progression of isolated nasal involvement in certain cases, and the surgery should be a last resort. Patients should be followed carefully over the long term since there is a tendency for recurrence and delayed systemic involvement.

## Competing interests

The author(s) declare that they have no competing interests.

## Authors' contributions

S.E. drafted and prepared the manuscript. S.S.E. reviewed the patient's medical record in order to collect all the available information. E.T. carried out the histopathologic evaluation. O.C. was involved in revising the article for intellectual content details. All authors read and approved the final manuscript.

## References

[B1] Heather R, Schwartz B, Tami T (2003). Ear nose and throat manifestations of sarcoidosis. Otolaryngol Clin North Am.

[B2] Shah UK, White JA, Gooey JE, Hybels RC (1997). Otolaryngologic manifestations of sarcoidosis presentations and diagnosis. Laryngoscope.

[B3] Braun JJ, Gentie A, Pauli G (2004). Sinonasal sarcoidosis; review and report of fifteen cases. Laryngoscope.

[B4] Wilson R, Lund V, Sweatman M, Mackay IS, Mitchell DN (1988). Upper respiratory tract involvement in sarcoidosis and its management. Eur Res J.

[B5] McCaffrey TV, McDonald TJ (1983). Sarcoidosis of the nose and paranasal sinuses. Laryngoscope.

[B6] Zeitlin JF, Tami TA, Brugman R, Winget D (2000). Nasal and sinus manifestations of sarcoidosis. Am J Rhinol.

[B7] Fergie N, Jones NS, Havlat MF (1999). The nasal manifestations of sarcoidosis: a review and report of eight cases. J Laryngol Otol.

[B8] Clark PC, Bondy P, Jacop L (2002). Radiology quiz case. Nasal sarcoidosis, in association with pulmonary sarcoidosis. Arch Otolaryngol Head Neck Surg.

[B9] Bourjat P, Braun JJ (2002). Sinonasal sarcoidosis: CT evaluation. J Radiol.

[B10] Krepsi YP, Kuriloff DB, Aner M (1995). Sarcoidosis of the sinonasal tract: a new staging system. Otolaryngol Head Neck Surg.

[B11] Long CM, Smith TL, Loehrl TA, Komorowski RA, Toohill RJ (2001). Sinonasal disease in patients with sarcoidosis. Am J Rhinol.

[B12] Marks S, Goodman R (1998). Surgical management of nasal and sinus sarcoidosis. Otolaryngol Head Neck Surg.

[B13] Kay DJ, Har-EL G (2001). The role of endoscopic sinus surgery in chronic sinonasal sarcoidosis. Am J Rhinol.

